# NR2F6-modified CAR T cells drive extrinsic immunogenic cell death and antigen-agnostic immunity in solid tumors

**DOI:** 10.1016/j.omton.2026.201189

**Published:** 2026-04-09

**Authors:** Victoria Klepsch, Dominik Wolf, Gottfried Baier

**Affiliations:** 1 Institute for Cell Genetics, Medical University of Innsbruck, Innsbruck, Austria; 2 Department of Internal Medicine V, Haematology & Oncology, Comprehensive Cancer Center Innsbruck and Tyrolean Cancer Research Institute, Medical University of Innsbruck, Innsbruck, Austria

## Main text

CAR T therapies have transformed selected blood cancers but continue to fall short in solid tumors,^1^ where antigen heterogeneity, antigen loss,^2^ and a profoundly immunosuppressive, myeloid-rich tumor immune microenvironment (TIME) blunt sustained therapeutic benefit.^3^^,^^4^^,^^5^ Building on our recent Nature Communications study,^6^ we now show that deleting the intracellular checkpoint NR2F6 does more than simply “supercharge” CAR T cells quantitatively. Instead, *Nr2f6*-deficient CAR T cells convert direct tumor cell killing into a robust, qualitatively distinct form of “extrinsic immunogenic cell death (ICD),” that is, ICD initiated by extrinsic immune effectors such as CAR T cells, which mobilizes the host immune system and supports durable, antigen-agnostic tumor control in solid tumors.

NR2F6 acts inside CD8^+^ CAR T cells as a brake on inflammatory and danger signaling when they enter the hostile TIME characteristic of solid tumors. In the presence of NR2F6, CAR T cells are held in check: their infiltration into tumor tissue is restricted, their effector cytokine production and cytolytic granule release are dampened, and their ability to ignite productive crosstalk with antigen-presenting cells is limited. Remarkably, however, removing this brake rewires how CAR T cells interpret and respond to suppressive cues: *Nr2f6*-deficient CAR T cells are significantly more resilient to chronic antigen exposure and inhibitory signaling within the TIME, while initiating a full ICD cascade that reshapes the tumor-immune dialogue.

Mechanistically, these engineered CAR T cells promote the release of damage-associated molecular patterns (DAMPs) from dying tumor cells, creating a danger-signal-rich TIME. This, in turn, enhances the uptake and cross-presentation of tumor antigens by dendritic cells (DCs). Activated DCs then prime and expand a polyclonal repertoire of endogenous CD8^+^ T cells, targeting not only the antigen recognized by the CAR but also additional tumor antigens uncovered through epitope spreading. In effect, CAR T cytotoxicity is repurposed into a potent *in situ* vaccination signal that enlists the broader host immune system into the therapy response.

The consequences of this shift are most evident in stringent solid tumor models characterized by pronounced immune suppression within the TIME and substantial antigen heterogeneity, where conventional CAR T cells generally achieve only transient disease control. In these models, standard CAR T products debulk antigen-positive tumor cells but rapidly succumb to antigen loss and to the cumulative inhibitory pressure of the TIME *in vivo*, leading to relapse. By contrast, *Nr2f6*-modified CAR T cells induce robust and durable tumor regressions that persist even after the infused CAR T cells have vanished from both the tumor and circulation. Tumor re-challenge experiments illustrate the change in response quality. After initial tumor clearance, mice treated with *Nr2f6*-deficient CAR T cells are protected against both antigen-matched and antigen-negative re-implanted tumors. This demonstrates that long-term antitumor control has been decoupled from continued CAR T persistence and from reliance on a single surface antigen. Instead, a broad endogenous T cell response, a kind of immune “memory portfolio” against multiple tumor antigens, maintains surveillance and clearance of residual or relapsing malignant cells, including those that have lost the original CAR target.

These data support a two-wave model of *Nr2f6*-targeted CAR T therapy in solid tumors. In the first wave, *Nr2f6*-deficient CAR T cells execute focused killing of antigen-positive tumor cells, like conventional CAR T products, but in a more inflammatory context. In the second wave, DCs interpret the resulting immunogenic debris, integrate DAMP-mediated danger signals, and prime endogenous CD8^+^ T cells against a broad spectrum of tumor antigens. The outcome is a polyclonal, antigen-diverse CD8^+^ T cell repertoire that is resilient to antigen heterogeneity and loss and capable of patrolling and eradicating residual disease. CD8^+^ T cell activation within the TIME thus emerges not as a binary on/off event but as a rheostat-like process in which NR2F6 helps integrate diverse extracellular cues via surface receptor dynamics, intracellular signaling cascades, and chromatin remodeling, thereby biasing this rheostat away from immune tolerance and toward host-protective antitumor immunity when NR2F6 is removed.

**Figure 1:**
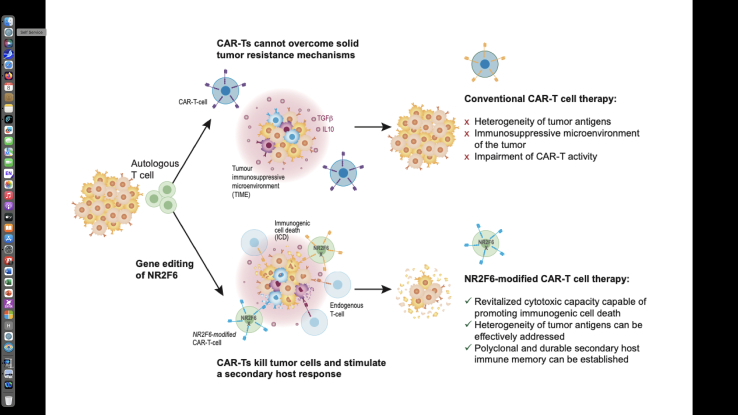
Enhanced therapeutic efficacy of NR2F6-targeted CAR T cells NR2F6-targeted CAR T cells in responders sustain cytotoxic activity, eradicate antigenically heterogeneous solid tumors, and elicit a durable, polyclonal host immune memory response.^6^ The overall concept is that the major limitations of conventional CAR T cells in solid tumors—antigen heterogeneity, a suppressive TIME, and limited persistence—are overcome by NR2F6-targeted CAR T cell design. In responders, these engineered cells retain sustained cytotoxic activity, effectively target heterogeneous tumor antigens, and provoke a durable, polyclonal host immune memory response. This approach demonstrates that antigenically diverse solid tumors can be efficiently eradicated, helping to bridge the efficacy gap between hematologic and solid malignancies in CAR T therapy. Together, these preclinical findings provide a rationale for further evaluation of NR2F6 as a potentially safe intracellular target to augment human CAR T therapy for solid tumors.

From the perspective of CAR T engineering, several messages emerge: first, achieving success in solid tumors with CAR T cells may require going beyond designs that simply boost direct cytotoxicity, proliferation, or persistence. The most effective products are likely to be those tuned to induce extrinsic ICD in tumor cells and to actively recruit host immunity, transforming CAR T cells from lone killers into orchestrators of multicellular antitumor responses. Second, NR2F6 functions as a tunable “checkpoint in the cell,” acting as an intracellular transcriptional gatekeeper that controls how CAR T cells integrate inhibitory cues and, via ICD, promote danger signals. As a ligandable orphan nuclear receptor, NR2F6 has been subjected to high-throughput small-molecule screening to identify synthetic modulators, suggesting that it could be drug-targetable.^7^ In turn, this raises the possibility that NR2F6 might be targeted both through genetic editing within CAR-expressing T cells and, in principle, through pharmacologic modulators, enabling future combination strategies aimed at fine-tuning this axis *in situ*. Third, the principles uncovered here (danger signaling, DC activation, and epitope spreading leading to polyclonal host T cell priming) may prove broadly applicable across CAR T platforms and disease indications, where antigen escape limits therapeutic durability. Whether the CAR targets a lineage antigen in hematologic malignancies or a tumor-associated antigen in solid tumors, the ability to convert each killing event into a vaccination-like stimulus could extend the host-protective depth and duration of antitumor immune responses. In this sense, *Nr2f6*-modified CAR T cells are a concrete example of a more general design principle: engineering CAR T products not only to kill more efficiently but to kill in a way that educates and amplifies the endogenous immune system (see Figure 1). More broadly, these preclinical findings argue for a shift in focus from surface-centric engineering toward rational rewiring of intracellular transcriptional checkpoints within CAR T cells to reshape the quality, not just the quantity, of their effector responses in complex TIMEs. Checkpoints such as NR2F6 are located at critical decision nodes that determine whether CAR T cells succumb to exhaustion, remain functionally restricted by suppressive myeloid cells, or instead generate the inflammatory and danger-signal-rich TIME needed for productive epitope spreading and systemic immunity. Targeting such nodes as NR2F6 may be particularly attractive in solid tumors, where simply adding co-stimulatory domains or cytokine payloads has not yet consistently overcome the multifactorial barriers to durable tumor control.^8^

By shifting the balance from a purely “kill” paradigm to a “kill-and-vaccinate” paradigm, *Nr2f6*-modified CAR T cells illustrate how next-generation designs might turn solid tumors from immune deserts into hubs of antigen-agnostic, systemic tumor immunity. As the field moves toward clinical translation of these concepts, NR2F6 provides both a mechanistic handle and a tractable target for reprogramming how CAR T cells communicate with and recruit the host immune system in solid tumors.

## Acknowledgments

The research was funded in part or in full by the Austrian Science Fund (FWF; grant-https://doi.org/10.55776/P31383 to G.B.., and grant-https://doi.org/10.55776/T1292 to V.K.), and the European Research Council Fund (ADG 786462, PoC 101054365, and PoC 101189004 to G.B.). Illustrations in the figure were created with BioRender, with artwork help from Delielena Poli; PNO Life Sciences & Health. For open-access purposes, the author has applied a CC BY public copyright license to any accepted version of the manuscript arising from this submission.

## Author contributions

All authors read, critically revised, and approved the final manuscript.

## Declaration of interests

The authors declare the following competing interests: K.V., D.W., and G.B. are inventors on patents related to immunological targets in the field of immuno-oncology.
